# Variation of lifespan in multiple strains, and effects of dietary restriction and *BmFoxO* on lifespan in silkworm, *Bombyx mori*

**DOI:** 10.18632/oncotarget.14235

**Published:** 2016-12-26

**Authors:** Jiangbo Song, Dongmei Tang, Zhiquan Li, Xiaoling Tong, Jianfei Zhang, Minjin Han, Hai Hu, Cheng Lu, Fangyin Dai

**Affiliations:** ^1^ State Key Laboratory of Silkworm Genome Biology, College of Biotechnology, Southwest University, Chongqing, China; ^2^ Key Laboratory for Sericulture Functional Genomics and Biotechnology of Agricultural Ministry, College of Biotechnology, Southwest University, Chongqing, China

**Keywords:** silkworm, lifespan, dietary restriction, BmFoxO, experimental animal, Gerotarget

## Abstract

Established animal models have accelerated our understanding of the mechanisms involved in lifespan determination. However, more experimental animals are required to clarify the complex mechanisms behind the phenomena of aging and lifespan. In this study, we reported the variation of lifespan in nine distinct silkworm strains. Lifespan correlated significantly with *BmFoxO* gene expression in the representative silkworm strains tested (Xiafang, Dazao-N, and N4). In general, the female silkworm was longer lived than the male of the same strain. Dietary restriction extended the silkworm lifespan compared with that of silkworms fed *ad libitum*. The expression of *BmFoxO* was significantly elevated in the dietary restriction group on day 3 of the 4^th^ instar and day 3 of the 5^th^ instar, suggesting that *BmFoxO* contributes to dietary restriction-mediated lifespan extension. The RNA interference and overexpression of the *BmFoxO* gene significantly shortened and extended the silkworm adulthood, respectively. In conclusion, our findings suggest that the silkworm might serve as a promising experimental animal to explore the complex biological mechanisms of lifespan determination.

## INTRODUCTION

Lifespan has fascinated many gerontologists for centuries. Previous studies have found that some lifespan regulation networks are conserved in various species ranging from invertebrates to vertebrates, including *Caenorhabditis elegans*, *Drosophila melanogaster*, and *Mus musculus* [1].

Experimental animals are an indispensable component of modern biomedical research, including molecular biology, translational medicine, and drug discovery, and also provide the fundamental basis and support for lifespan studies. The increasing number of animal models have been established for studies of aging and lifespan. However, the existing animal models do not fully meet the needs of complex lifespan studies, and in particular, there is an acute shortage of experimental invertebrate animal model (which currently predominantly include flies and worms) [2–4]. Therefore, new experimental invertebrate animals are required to explore the lifespan decision mechanism.

Silkworm was a classical experimental animal in genetic research in the 20^th^ century, during which it played a vital role comparable with *Drosophila* [5–7]. The vast amount of silkworm genetic resources available still provides important material for biological studies [5, 6]. Silkworm has numerous beneficial traits making it a potential model organism for lifespan research, such as clear genetic background, moderate body size, appropriate life cycle, high propagation rate, and clear boundaries of developmental stages. In *Drosophila melanogaster*, the number of larval instars appears to be firmly fixed at three [8], whereas in silkworm, the number of larval instars is approximately five (three to six), indicating its greater plasticity in lifespan extension. Several advances in lifespan research have been achieved in the silkworm, making it an increasingly appropriate experimental animal in this field [9, 10].

In this study, we conducted the survey of lifespan in nine strains and evaluated the impact of dietary restriction (DR) on lifespan. We also detected the expression of *BmFoxO*, which encodes an evolutionarily conserved integrator in lifespan regulation networks. We preliminarily investigated the relationship between *BmFoxO* and lifespan using *BmFoxO*-specific RNA interference (RNAi) and *BmFoxO* overexpression in silkworm.

## RESULTS

### Significant lifespan variation among different silkworm strains

**Figure 1:**
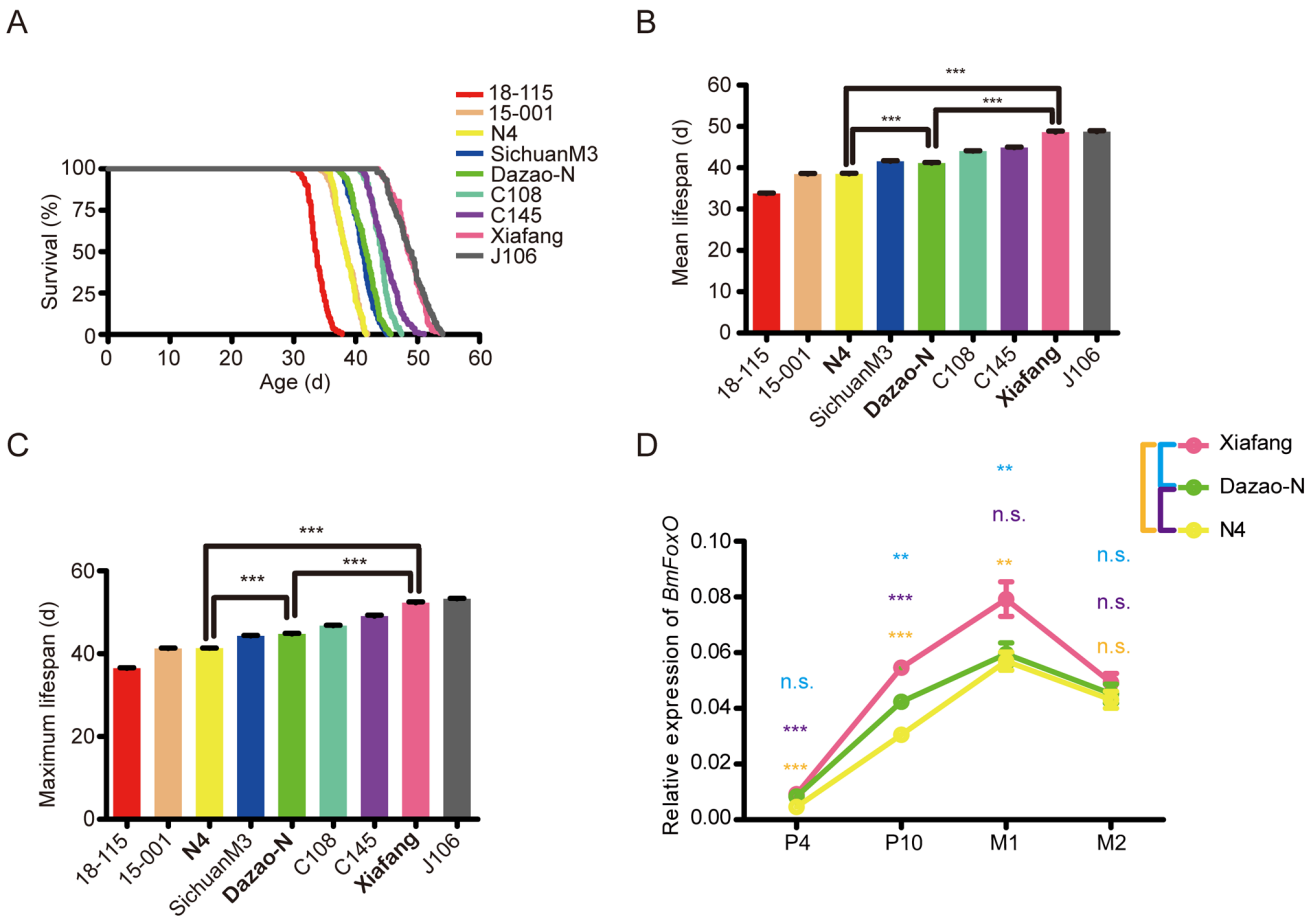
Mean lifespan, maximum lifespan, and *BmFoxO* expression in different silkworm strains **A**. Survival curves for silkworm strains 18-115, 15-001, N4, SichuanM3, Dazao-N, C108, C145, Xiafang, and J106. **B**. Mean lifespan of the nine strains. **C**. Maximum lifespan of the nine strains. **D**. Expression of *BmFoxO* at P4 (day 4 of pupa), P10 (day 10 of pupa), M1 (day 1 of moth), and M2 (day 2 of moth) in three representative strains.

To determine whether there are some suitable strains for lifespan studies in silkworm, the lifespan of nine silkworm strains was surveyed, and the moment of different developmental nodes (hatching, pupation, eclosion, and death) were recorded. A statistical analysis indicated that the lifespan of the nine strains differed significantly. J106 was the most long-lived strain (48.96 days, n = 138), followed by Xiafang (48.46 days, n = 123), C145 (44.83 days, n = 161), C108 (44.08 days, n = 121), Dazao-N (41.71 days, n = 182), SichuanM3 (41.08 days, n = 139), N4 (38.42 days, n = 127), 15-001 (38.46 days, n = 172), and 18-115 (33.65 days, n = 194), which was the relatively short-lived strain. The Kaplan-Meier mortality curve, mean lifespan, and maximum lifespan of each strain are shown in Figure 1A-1C. The results of significance analyses of mean lifespan and maximum lifespan are presented in Table S2 and S3, respectively.

To determine whether *BmFoxO* plays an important role in the lifespan difference of these strains, three representative strains (Xiafang, relatively long-lived; Dazao-N, wild type; N4, relatively short-lived) were selected to test the correlation between *BmFoxO* expression and lifespan. Results showed that Xiafang was the longest-lived strain, followed by Dazao-N, and N4 was the shortest-lived strain (Figure 1A-1C), and the expression of *BmFoxO* was highest in Xiafang, followed by Dazao-N, and then N4 (Figure 1D). Surprisingly, we detected positive correlations among *BmFoxO* expression, mean lifespan, and maximum lifespan in all four developmental stages of the three tested strains (Figure 1B-D).

**Figure 2:**
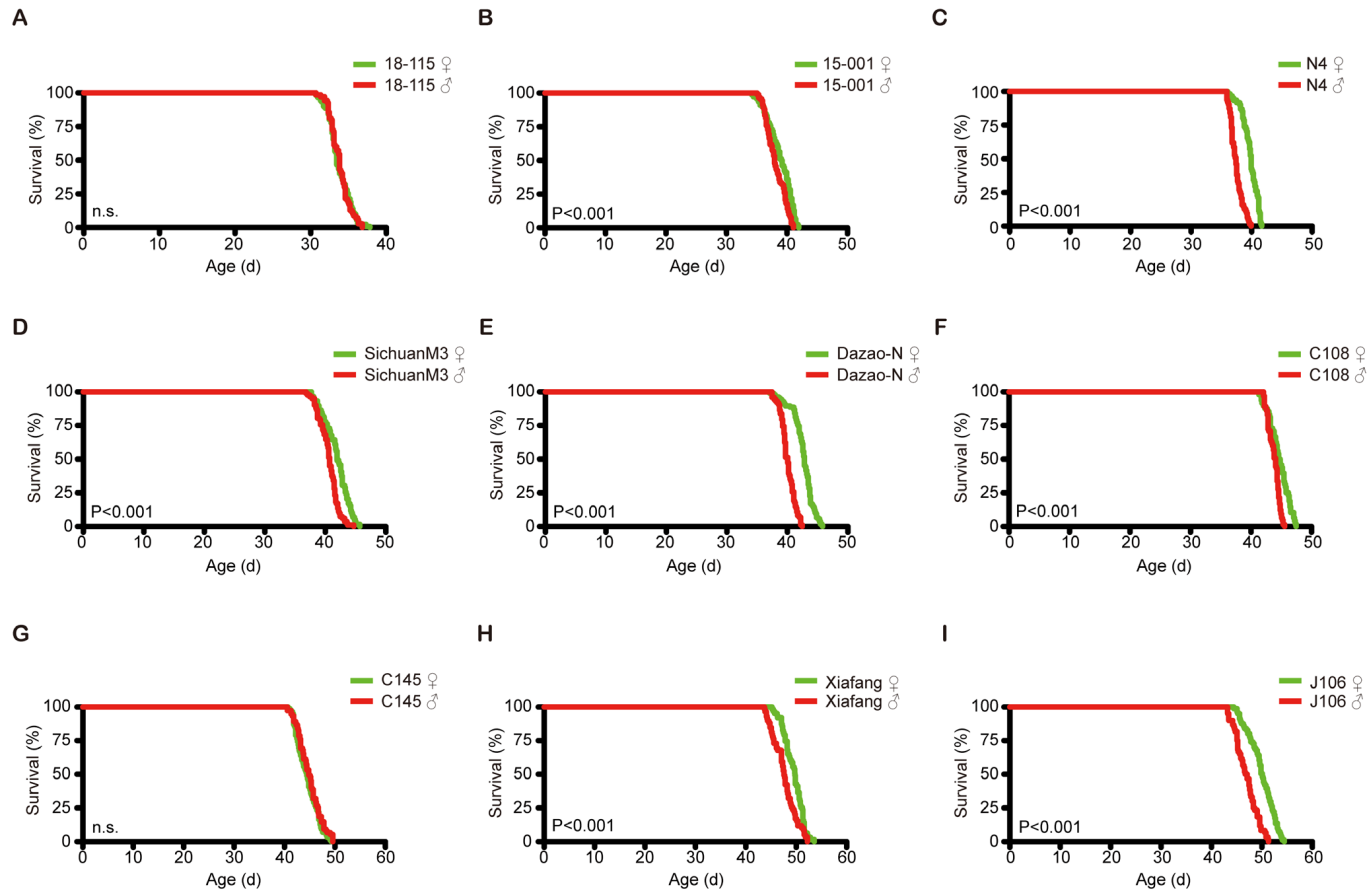
Lifespan differences between males and females in each of nine strains A Log-rank (Mantel-Cox) test was used to evaluate the difference between the male and female lifespan in each strain. **A**. 18-115; **B**. 15-001; **C**. N4; **D**. SichuanM3; **E**. Dazao-N; **F**. C108; **G**. C145; **H**. Xiafang; **I**. J106.

In a variety of species, female animals are generally longer lived than males. To determine whether the silkworm lifespan displays this sexual dimorphism, we compared the lifespan of male and female silkworms in each of the nine strains. The lifespan of the female was longer than that of the male in all the tested strains, except 18-115 and C145, which is consistent with the conservation of this characteristic throughout the animal kingdom (Figure 2).

### Lifespan extension in silkworm under dietary restriction

**Figure 3:**
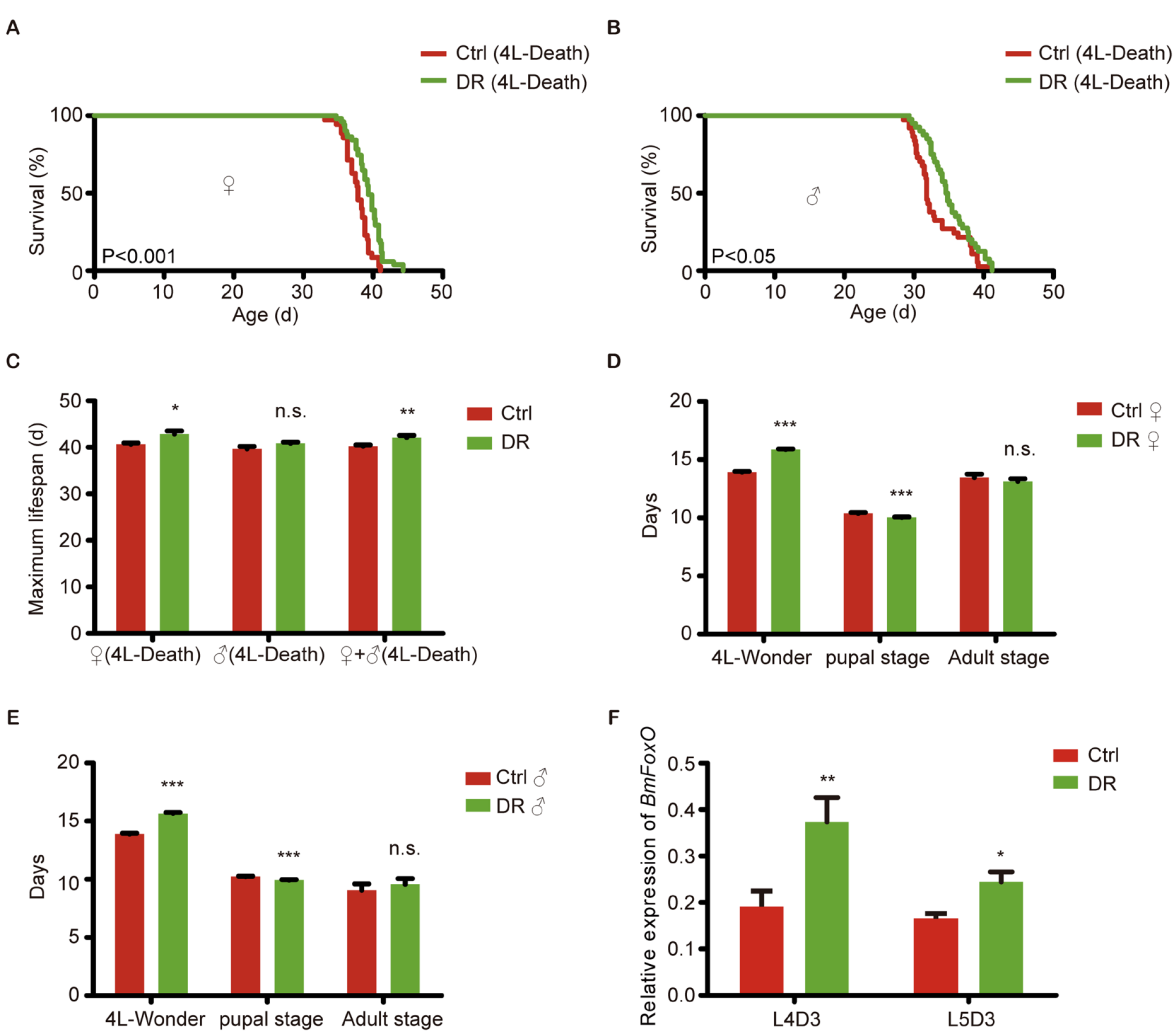
Percentage survival of DR-treated silkworms **A**. Survival curves for the female DR groups and controls. **B**. Survival curves for the male DR groups and controls. **C**. Maximum lifespan of the DR groups and controls. **D**. Comparison of the female DR groups and controls in different developmental stages. **E**. Comparison of the male DR groups and controls in different developmental stages. **F**. Relative expression of *BmFoxO* in the DR groups and controls at L4D3 and L5D3. Error bar represents the SEM.

Dietary restriction extends the animal's lifespan in plenty of animal models [11–14]. To examine the effects of DR on the silkworm lifespan and its mechanism, we performed a lifespan expectancy test of DR in the wild-type Dazao-N strain from 4^th^ instar. The mean lifespan of the females in the DR group was 39.35 days, from the 4^th^ instar to death, whereas that of the female controls was 37.67 days (increased by 4.46%) (Figure 3A). In males, the mean lifespan of the DR group was 35.15 days, and that of the controls was 33.18 days (increased by 5.94%) (Figure 3B). The maximum lifespan was 4.72% longer in the DR group than in the control for both sexes combined (Figure 3C). Under DR, the larval stage and pupal stage were prolonged and shortened, respectively, whereas the adult stage has not been altered (Figure 3D-3E). *BmFoxO* expression also increased significantly in the DR group on day 3 of the 4^th^ instar (L4D3) and day 3 of the 5^th^ instar (L5D3) (Figure 3F). These results indicate that DR prolongs the silkworm lifespan which might be mediated by *BmFoxO* increase.

### BmFoxO contributes to silkworm lifespan extension

**Figure 4:**
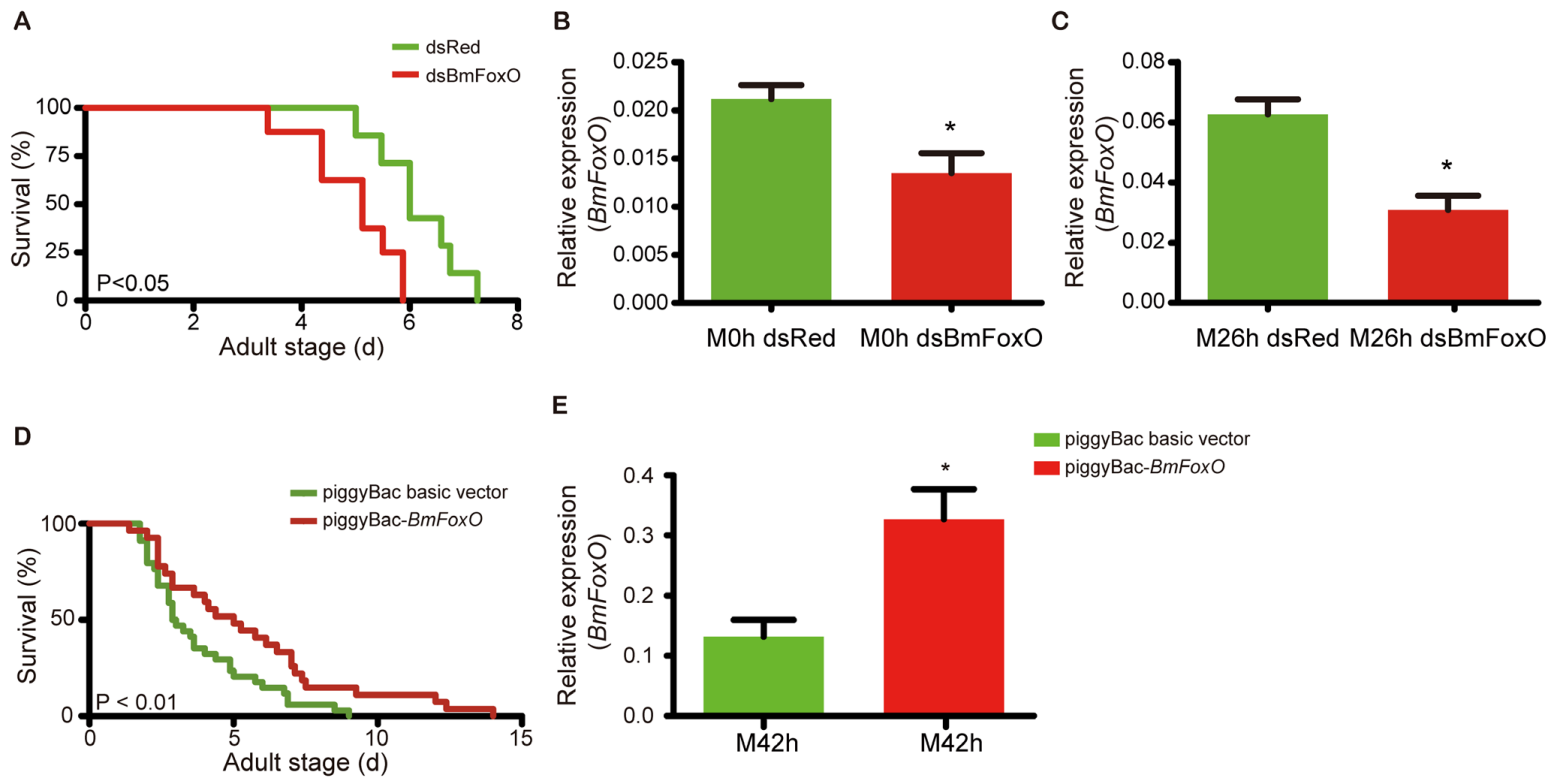
*BmFoxO* RNA interference and overexpression **A**. Survival of adulthood after injection of dsBmFoxO or dsRed. **B**. Relative expression of *BmFoxO* at 0 hour after eclosion. **C**. Relative expression of *BmFoxO* at 26 hour after eclosion. **D**. Survival of adult stage in silkworms injected with piggyBac-*BmFoxO* or piggyBac basic vector. **E**. Relative expression of *BmFoxO* at 42 hour after eclosion. *p < 0.05, **p < 0.01, ***p < 0.001.

To investigate the influence of *BmFoxO* on the lifespan of the silkworm, RNA interference based knockdown was performed. Based on the spatiotemporal expression profile of *BmFoxO* in the silkworm (Figure S1), *BmFoxO* double-stranded RNA (dsRNA) was injected into the hemolymph at day 10 of the pupal stage, one day before the peak in *BmFoxO* expression. We demonstrated that the shortened silkworm lifespan resulted from a reduction of *BmFoxO* expression, which was confirmed by its quantification at 0 hour and 26 hour after eclosion (Figure 4A-4C).

To determine the effect of *BmFoxO* overexpression on the silkworm lifespan, we injected *BmFoxO* expression plasmid (piggyBac-*BmFoxO*) or a control plasmid (piggyBac basic) into the silkworm hemolymph. The result showed that the adult stage was prolonged (by 43%) following an increase in the expression of *BmFoxO* at 42 hour after eclosion (Figure 4D-4E, Figure S2).

## DISCUSSION

With the lack of invertebrate models for lifespan studies, new model invertebrates are urged to be identified. Almost 700 strains are maintained in the Silkworm Gene Bank in Southwest University, China, which have potential utility in the exploration of complicated lifespan mechanisms. New models could also be generated using genome-editing tools. Furthermore, for transient functional studies, gene expression manipulation (overexpression or RNAi) and pharmacological interventions (small-molecule drugs) can be readily achieved with injection or feeding [15]. Therefore, insight into complex lifespan mechanisms could be gained by the application of discovered suitable natural resources and well-established artificial models.

In this study, the silkworm lifespan was extended under DR condition. DR mediated a prolonged larval stage, shortened pupal stage, and unaltered adult stage of the silkworm. A possible explanation is that silkworms only feed in the larval stage, and once the treatment ceases, the beneficial effects of DR are weakened until completely disappear. The benefits accumulated in the larval stage may facilitate the pupae development. Our study suggests that *BmFoxO* plays a potent role in DR-mediated lifespan extension, which is consistent with the roles of *Drosophila dFoxO* and nematode *DAF-16*, orthologs of silkworm *BmFoxO*, in the beneficial effects of DR [12, 16].

Our finding that *BmFoxO* expression exactly contributes the silkworm lifespan extension is consistent with the previous studies in other species [1, 17–19], and support the potential application utility of the silkworm as an animal model in clarifying the *FoxO*-mediated lifespan decision process. In addition, the further identification and characterization of the downstream targets of *BmFoxO* will also provide new insight into the function of *FoxO* in the lifespan determination [20–23].

## MATERIALS AND METHODS

### Silkworm strains and rearing conditions

Nine silkworm strains (univoltinism: C145, J106; bivoltinism:18-115, 15-001, SichuanM3, Dazao-N, C108, Xiafang; multivoltinism: N4) were reared at the Silkworm Gene Bank in Southwest University and maintained in standard conditions (25 °C, in approximately 75% relative humidity with a 12L: 12D photoperiod) [9] and were reared with clean and fresh mulberry leaves during the whole larval stage.

### Lifespan assay

For the lifespan assays, silkworm were raised in incubators during the whole life cycle. For making accurate estimation of mean lifespan, each of the silkworms is reared in a separate cage and observed every 3 hours. The survivorship of each moth was recorded once died.

### DR treatment in silkworm

Silkworm, Dazao-N strain, was used as a material for investigating the relationship between DR and lifespan. The control and treatment group silkworms were fed with fresh mulberry leaves at 100% and 60% of the *ad libitum* feeding from the beginning of 4^th^ instar larvae respectively. Temperature, humidity and photoperiod were under the standard conditions [9].

### RNA extraction and real-time quantitative PCR (RT-qPCR)

The control and treatment silkworms were obtained for RNA extraction and RT-qPCR. A rapid extraction Total RNA Kit (BioTeke, China) was employed for total RNA extraction of three individuals in Trizol reagent (Invitrogen, USA) according to the manufacturer's instructions. The first strand cDNA was synthesized from the total RNA samples with the PrimeScript^TM^ RT Reagent Kit including gDNA Eraser (TaKaRa, Japan).

The RT-qPCRs were performed on a CFX96 Real-Time System (Bio-Rad, USA) with an iTaq Universal SYBR Green Supermix (Bio-Rad, USA), according to the manufacturer's recommended procedure. The sw22934 (BmMDB probe ID), an optimal stable gene in silkworm, was used as the internal control [24]. Primer pairs targeted to the regions of sw22934 and *BmFoxO* gene specifically (Table S1). The RT-qPCR conditions followed the manufacturer's instructions.

### RNA interference of BmFoxO at moth stage

To perform RNAi of *BmFoxO*, a dsRNA fragment was obtained by PCR amplification from the full-length *BmFoxO* cDNA with a pair of primers containing T7 RNA polymerase-binding site (Table S1). The *BmFoxO* dsRNA (dsBmFoxO) and the red fluorescent protein gene dsRNA (dsRed) were synthesized using T7 RiboMAX Express RNAi kit (Promega, USA) according to the guidance instructions. The dsRNA (120 μg) was injected into hemolymph through the penultimate spiracle of silkworm at the 10^th^ day after pupation [25]. Lifespan data was obtained after dsRed and dsBmFoxO treatments. Samples were collected for RT-qPCR analysis at 0 hour and 26 hour after molting.

### Cloning, vector constructs and BmFoxO overexpression at moth stage

The sequence, containing the complete immediate-early gene (IE1) promoter, *BmFoxO* cDNA, and the SV40 late PolyA signal, was inserted into the multiple cloning site of piggyBac basic vector to generate the piggyBac-*BmFoxO* plasmid, which was used as the overexpression vector. The piggyBac basic plasmid was used as the control. Each vector (5 μg) was injected into hemolymph through the penultimate spiracle of silkworm (Dazao-N) at the 10^th^ day after pupation.

### Statistics

The statistical analysis was performed with the GraphPad Prism (GraphPad Software, USA). The Kaplan-Meier curve was illustrated and Log-rank (Mantel-Cox) test was employed to estimate the statistical differences of survival between treatments and controls using GraphPad Prism 5. Student's *t* test and two-tailed unpaired *t* test were employed for the significance analysis of histogram, * p < 0.05; ** p < 0.01; *** p < 0.001. Means were calculated from at least three independent individuals.

## SUPPLEMENTARY MATERIALS TABLES FIGURES









## Abbreviations

DR: Dietary restriction
RNAi: RNA interference
dsRNA: double-stranded RNA
