# Supplementary material for: Variation of lifespan in multiple strains, and effects of dietary restriction and BmFoxO on lifespan in silkworm, Bombyx mori
Source: Oncotarget. 2016 Dec 26;8(5):7294–300. doi: 10.18632/oncotarget.14235 (PMC5352321; doi:10.18632/oncotarget.14235)
Supplement: Supplementary file 1 [file oncotarget-08-7294-s001.pdf]

Variation of lifespan in multiple strains, and effects of dietary restriction and *BmFoxO* on lifespan in silkworm, *Bombyx mori*

Supplementary Material

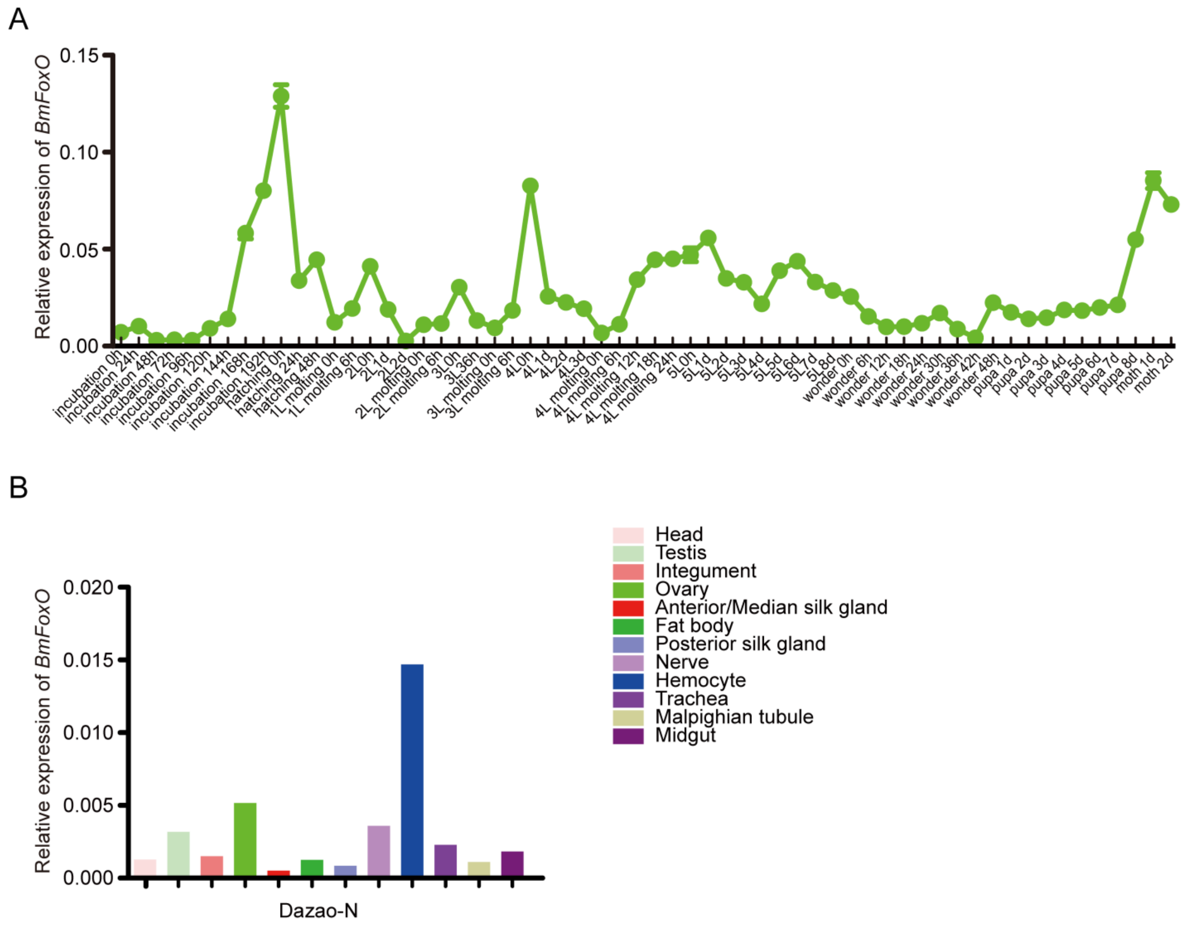

Figure S1: Spatiotemporal expression profile of *BmFoxO* in Dazao-N strain.

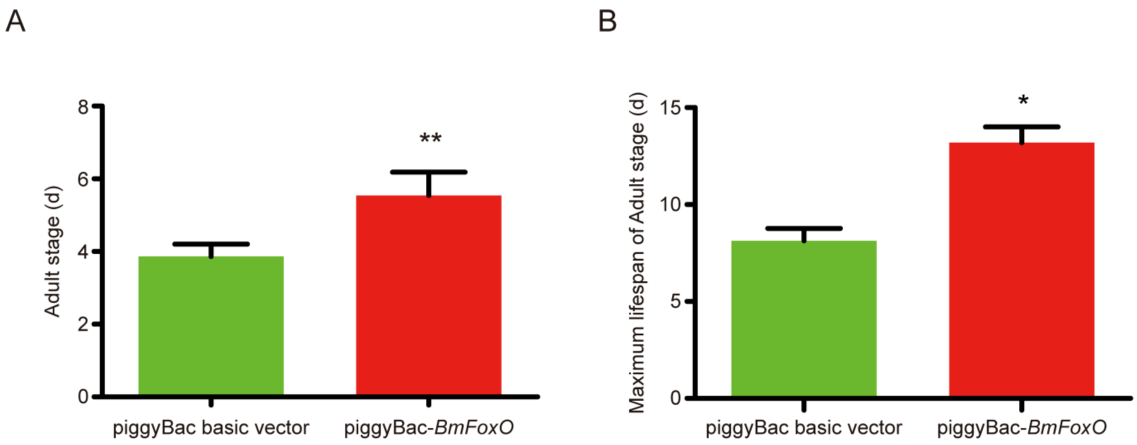

Figure S2: Mean and maximum lifespan of adult stage after *BmFoxO* overexpression.
